# Assessment of 4D flow MRI's quality by verifying its Navier–Stokes compatibility

**DOI:** 10.1002/cnm.3603

**Published:** 2022-05-09

**Authors:** Jeremías Garay, Hernán Mella, Julio Sotelo, Cristian Cárcamo, Sergio Uribe, Cristóbal Bertoglio, Joaquín Mura

**Affiliations:** ^1^ Bernoulli Institute University of Groningen Groningen The Netherlands; ^2^ Biomedical Imaging Center Pontificia Universidad Católica de Chile Santiago Chile; ^3^ Millennium Nucleus in Cardiovascular Magnetic Resonance, Cardio MR Santiago Chile; ^4^ Department of Electrical Engineering Pontificia Universidad Católica de Chile Santiago Chile; ^5^ School of Biomedical Engineering Universidad de Valparaiso Valparaiso Chile; ^6^ Millennium Institute for Intelligent Healthcare Engineering, iHEALTH Santiago Chile; ^7^ Department of Mathematical Engineering Universidad de Concepción Concepción Chile; ^8^ Institute for Biological and Medical Engineering, Schools of Engineering, Medicine and Biological Sciences Pontificia Universidad Católica de Chile Santiago Chile; ^9^ Department of Radiology, Schools of Medicine Pontificia Universidad Católica de Chile Santiago Chile; ^10^ Department of Mechanical Engineering Universidad Técnica Federico Santa María Santiago Chile

**Keywords:** 4D flow MRI, blood flows, Navier–Stokes equations, stabilized finite elements

## Abstract

4D Flow Magnetic Resonance Imaging (MRI) is the state‐of‐the‐art technique to comprehensively measure the complex spatio‐temporal and multidirectional patterns of blood flow. However, it is subject to artifacts such as noise and aliasing, which due to the 3D and dynamic structure is difficult to detect in clinical practice. In this work, a new mathematical and computational model to determine the quality of 4D Flow MRI is presented. The model is derived by assuming the true velocity satisfies the incompressible Navier–Stokes equations and that can be decomposed by the measurements ${\vec{u}}_{\text{meas}}$ plus an extra field $\vec{w}$. Therefore, a non‐linear problem with $\vec{w}$ as unknown arises, which serves as a measure of data quality. A stabilized finite element formulation tailored to this problem is proposed and analyzed. Then, extensive numerical examples—using synthetic 4D Flow MRI data as well as real measurements on experimental phantom and subjects—illustrate the ability to use $\vec{w}$ for assessing the quality of 4D Flow MRI measurements over space and time.

## Graphical abstract

A new mathematical and computational model to determine the quality of 4D Flow MRI is presented. The model is derived by assuming the true velocity satisfies the incompressible Navier–Stokes equations and that it can be decomposed by the measurements plus an error field *w*. Therefore, a problem for *w* arises which serves as a measure of data quality.

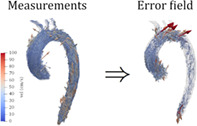

## 1 INTRODUCTION

Time‐resolved 3D flow magnetic resonance imaging, known as 4D Flow MRI, has shown in the last years potential in assessing cardiovascular diseases since it offers full coverage of the region of interest, therefore allowing for its analysis after the scan.^1^, ^2^ Additionally, it allows the computation of several hemodynamic parameters, which can be used as new biomarkers.^2^ However, high‐quality 4D Flow in subjects involves long time scans (>20 min) even with coarse spatio‐temporal resolutions making it challenging for everyday clinical use. In order to accelerate the acquisition time, several strategies have been proposed, such as parallel imaging^3^, ^4^ which accelerates the acquisition by exploiting the sensitivity of multiple receivers, and k‐space undersampling^5^, ^6^, ^7^, ^8^ which exploits data redundancies in frequency and time. This scan time reduction comes at the price of reducing the signal‐to‐noise ratio (SNR). SNR also decreases when reducing the image's voxel size. Moreover, the velocity field can only be obtained under a certain predefined range which depends on the magnetic gradient setup, therefore being potentially subject of velocity aliasing. Other artifacts may also appear due to subject's respiration and motion during the scan.

To the best of the author's knowledge, quality control of 4D Flow in clinics is based on calculation of peak/mean flows, mean velocities, flow patterns, and stroke volumes.^2^, ^9^, ^10^ A more systematic approach is to compute the divergence field of the data: assuming the blood flow is incompressible, jumps in the divergence field may indicate the presence of artifacts. Indeed, the incompressibility assumption has been used for denoising^9^, ^11^, ^12^, ^13^ and as a regularization term during the reconstruction process.^14^, ^15^ However, an important limitation of the divergence is that any measured velocity will have “infinitely” large divergence compared to its reference value which would be zero. Therefore, to the best of our understanding, only the spatial distribution of the divergence may be used as an error indicator but not in absolute terms for image quality check. Moreover, it will be shown later in the article, measurement artifacts may lead to no important changes in the divergence field.

Therefore, this work introduces an alternative quantitative approach for assessing 4D Flow quality by verifying the compatibility with the linear momentum conservation part of the Navier–Stokes, which when being written appropriately, includes also angular momentum and mass conservation.

The rest of this article is structured as follows. In Section 2, the mathematical model will be introduced, and a numerical method will be developed and analyzed. In Section 3, a set of relevant examples, using synthetic data, will be detailed. Numerical computations of the new model for several types of artifacts and its comparison against the divergence of the data are also shown. Results with phantom and subject's 4D Flow MRI are shown in Section 4. Finally, in Section 5, we discuss potential applications of this metric in the context of reconstruction and processing 4D Flow MRI.

## 2 THE MATHEMATICAL MODEL

### 2.1 The continuous problem

We assume a physical velocity field $\vec{u}$, which satisfies the conservation of linear momentum of the incompressible Navier–Stokes equations in the vessel lumen Ω:

(1) $$\rho \frac{\partial \vec{u}}{\partial t}+\rho \left(\vec{u}\cdot \nabla \right)\vec{u}-\mu \Delta \vec{u}+\nabla p=0\;\text{in}\;\Omega ,$$

where p is the fluid pressure field, and ρ and μ are the density and dynamic viscosity of the fluid, respectively. We recall that both the convective representation of the advection term and Laplacian representation of the viscous term are written in a simplified form using that $\nabla \cdot \vec{u}=0$. Moreover, the constitutive model (incompressible Newtonian model) represented by the stress terms (viscous plus pressure) is derived by enforcing conservation of angular momentum.

Let us denote ${\vec{u}}_{\text{meas}}$ the 4D Flow measurement field. We assume that there exist a field $\vec{w}$, that satisfies:

(2) $$\vec{u}={\vec{u}}_{\text{meas}}+\vec{w}\;\text{in}\;\Omega$$

(3) $$\nabla \cdot \vec{w}=0\;\text{in}\;\Omega$$

(4) $$\vec{w}=\vec{0}\;\mathrm{on}\;\partial \Omega ,$$

with ∂Ω being the whole boundary of Ω. By writing (1) in weak form, and using relations ((2), (3), (4)), we can formulate the following weak problem: Find $\left(\vec{w}\left(t\right),p\left(t\right)\right)\in H_{0}^{1}\left(\Omega \right)\times L_{0}^{2}\left(\Omega \right)$ such that

$$\int_{\;\Omega }\rho \frac{\partial \vec{w}}{\partial t}\cdot \vec{v}+\rho \left(\left({\vec{u}}_{\text{meas}}+\vec{w}\right)\cdot \nabla \right)\vec{w}\cdot \vec{v}+\rho \left(\vec{w}\cdot \nabla \right){\vec{u}}_{\text{meas}}\cdot \vec{v}+\mu \nabla \vec{w}:\nabla \vec{v}-p\nabla \cdot \vec{v}+q\nabla \cdot \vec{w}$$

(5) $$=-\int_{\;\Omega }\rho \frac{\partial {\vec{u}}_{\text{meas}}}{\partial t}\cdot \vec{v}+\rho \left({\vec{u}}_{\text{meas}}\cdot \nabla \right){\vec{u}}_{\text{meas}}\cdot \vec{v}+\mu \nabla {\vec{u}}_{\text{meas}}:\nabla \vec{v}$$

for all $\left(\vec{v},q\right)\in H_{0}^{1}\left(\Omega \right)\times L_{0}^{2}\left(\Omega \right)$.

The following remarks are in order.

Remark 1 The left‐hand‐side of Problem (5) resembles the incompressible Navier–Stokes equation, up to two additional terms. Unfortunately, none of these terms are (semi‐)positive‐definite. Therefore, the analysis of the well posedness of this continuous problem (existence, uniqueness, time‐stability) becomes challenging. However, those properties can be ensured at the discrete level by including adequate stabilization terms and constraints on the physical constants.

Remark 2 Equation (1) uses the so‐called convective form of the advective term. While alternative forms of Equation (5) could be derived starting from other forms for the advection (e.g., conservative), the resulting discrete problem will need to be stabilized to become a solvable problem, leading to the same expression for the bilinear form. There will be, however, a difference in right‐hand‐side terms. There is, however, no particular reason to choose one above the other since all formulations are consistent with perfect (i.e., divergence‐free) measurements.

Remark 3 An alternative formulation can be introduced by re‐defining Equation (3) as

(6) $$\nabla \cdot \vec{w}=-\nabla \cdot {\vec{u}}_{\text{meas}}+\frac{1}{|\Omega |}\int_{\partial \Omega }{\vec{u}}_{\text{meas}}\cdot \vec{n},$$

where the second term in the right‐hand‐side is needed to enforce the compatibility with respect to the boundary condition (4). However, as will be show later on, this leads $\vec{w}$ to have in general larger values than using the divergence‐free model, even in places with no or little measurement errors, reducing its potential utility.

Remark 4 The regularity assumptions required to solve for $\vec{w}$ are stronger than the usual perturbations in real data, which are often not additive in nature and present strong jumps in space and time. Therefore, we do not aim that $\vec{w}$ is capable of “correcting” the data (e.g., by computing ${\vec{u}}_{\text{meas}}+\vec{w}$) as it may be the purpose of, for example, data assimilation approaches. The field $\vec{w}$ rather aims to provide a complementary metric to detect defects in the measurements.

Remark 5 The choice of homogeneous Dirichlet boundary conditions is based on its consistency with the case of perfect measurements. In contrast, natural boundary conditions are not consistent: if $\vec{w}=\vec{0}$ everywhere the pressure p cannot become the physical pressure on boundaries where homogenous Neumann boundary condition is enforced.

Remark 6 Note that the so‐called “Stokes estimator” (STE) method for pressure reconstruction^16^ is recovered dropping the first three terms of the left‐hand‐side.

### 2.2 Stabilized finite element formulation

The tetrahedral mesh with characteristic element size h obtained from the segmented medical image is denoted by $\Omega_{h}$, which is the discrete domain over we define the following functions spaces

$$V_{h}=\left\{\vec{w}\in {\left[H_{0}^{1}\left(\Omega_{h}\right)\right]}^{3}:\vec{w}\in {\left[{\mathbb{P}}_{1}\left(K\right)\right]}^{3}\forall K\in \Omega_{h}\right\}$$

and

$$Q_{h}=\left\{q\in L_{0}^{2}\left(\Omega_{h}\right)\cap H^{1}\left(\Omega_{h}\right):q\in {\mathbb{P}}_{1}\left(K\right)\forall K\in \Omega_{h}\right\}.$$

In order to aim for the clinical applicability, it is crucial to use fast and robust numerical schemes. For the spatial discretization, we adopt $V_{h}$ and $Q_{h}$ as spaces for $\vec{w}$ and p, respectively, using stabilized finite elements to ensure solvability. For the time discretization, we consider a backward Euler method with fixed time step τ to avoid GCL‐type conditions. In order to avoid a root‐finding problem at each time step the non‐linear term on $\vec{w}$ will be treated semi‐implicitly.

The resulting fully discrete stabilized formulation reads as follows. Given ${\vec{w}}^{0}=\vec{0}$, for k≥1 find $\left({\vec{w}}^{k},p^{k}\right)\in V_{h}\times Q_{h}$ such that

(7) $$B^{k}\left({\vec{w}}^{k},p^{k};\vec{v},q\right)={ℒ}^{k}\left(\vec{v},q\right)$$

for all $\left(\vec{v},q\right)\in V_{h}\times Q_{h}$. The stabilized bilinear form is defined as:

(8) $$B^{k}\left(\vec{w},p;\vec{v},q\right)≔A^{k}\left(\vec{w},p;\vec{v},q\right)+S_{\text{conv}}^{k}\left(\vec{w};\vec{v}\right)+S_{\text{press}}^{k}\left(\vec{w},p;\vec{v},q\right)$$

with

(9) $$A^{k}\left(\vec{w},p;\vec{v},q\right)≔\int_{\;\Omega }\frac{\rho }{\tau }\vec{w}\cdot \vec{v}+\rho \left(\left({\vec{u}}_{\text{meas}}^{k}+{\vec{w}}^{k-1}\right)\cdot \nabla \right)\vec{w}\cdot \vec{v}+\rho \left(\vec{w}\cdot \nabla \right){\vec{u}}_{\text{meas}}^{k}\cdot \vec{v}+\mu \nabla \vec{w}:\nabla \vec{v}-p\nabla \cdot \vec{v}+q\nabla \cdot \vec{w}$$

being the bilinear form associated to the non‐stabilized weak form of (5), while the convection stabilization term is given by

$$S_{\text{conv}}^{k}\left(\vec{w};\vec{v}\right)≔\int_{\;\Omega }\frac{\rho }{2}\;\left(\nabla \cdot \left({\vec{u}}_{\text{meas}}^{k}+{\vec{w}}^{k-1}\right)\right)\;\vec{w}\cdot \vec{v}$$

and the pressure stabilization term as

$$S_{\text{press}}^{k}\left(\vec{w},p;\vec{v},q\right)≔\frac{\delta h^{2}}{\mu }\;\int_{{\;\Omega }_{h}}\;\left(\rho \left(\left({\vec{u}}_{\text{meas}}^{k}+{\vec{w}}^{k-1}\right)\cdot \nabla \right)\vec{w}+\rho \left(\vec{w}\cdot \nabla \right){\vec{u}}_{\text{meas}}^{k}+\nabla p\right)\cdot \left(\rho \left(\left({\vec{u}}_{\text{meas}}^{k}+{\vec{w}}^{k-1}\right)\cdot \nabla \right)\vec{v}+\rho \left(\vec{v}\cdot \nabla \right){\vec{u}}_{\text{meas}}^{k}+\nabla q\right)$$

with δ>0 some user‐defined parameter. Finally, the right‐hand‐side is given by

$${ℒ}^{k}\left(\vec{v},q\right)≔\int_{\;\Omega }\frac{\rho }{\tau }{\vec{w}}^{k-1}\cdot \vec{v}+\ell^{k}\left(\vec{v},q\right)$$

with

$$\ell^{k}\left(\vec{v},q\right)≔\int_{\;\Omega }{\vec{f}}^{k}\cdot \vec{v}-\mu \nabla {\vec{u}}_{\text{meas}}^{k}:\nabla \vec{v}+\frac{\delta h^{2}}{\mu }\int_{\Omega_{h}}{\vec{f}}^{k}\cdot \left(\rho \left(\left({\vec{u}}_{\text{meas}}^{k}+{\vec{w}}^{k-1}\right)\cdot \nabla \right)\vec{v}+\rho \left(\vec{v}\cdot \nabla \right){\vec{u}}_{\text{meas}}^{k}+\nabla q\right)$$

and ${\vec{f}}^{k}=-\rho \left({\vec{u}}_{\text{meas}}^{k}-{\vec{u}}_{\text{meas}}^{k-1}\right)/\tau -\rho \left({\vec{u}}_{\text{meas}}^{k}\cdot \nabla \right){\vec{u}}_{\text{meas}}^{k}$. Additional theoretical properties of Problem (5) in terms of its solvability and time stability of the solution are shown in Appendix A.

Remark 7 The stabilization term $S_{\text{conv}}^{k}$ is in general not consistent with the solution of (5). However, it is consistent when the measurements are perfect since, in such case $\vec{w}=0$ and $\nabla \cdot {\vec{u}}_{\text{meas}}=0$. The stabilization term $S_{\text{press}}^{k}$ is weakly consistent due to the inclusion of $h^{2}$.

## 3 NUMERICAL EXPERIMENTS USING SYNTHETIC DATA

### 3.1 Procedure for synthetic data generation

**FIGURE 1:**
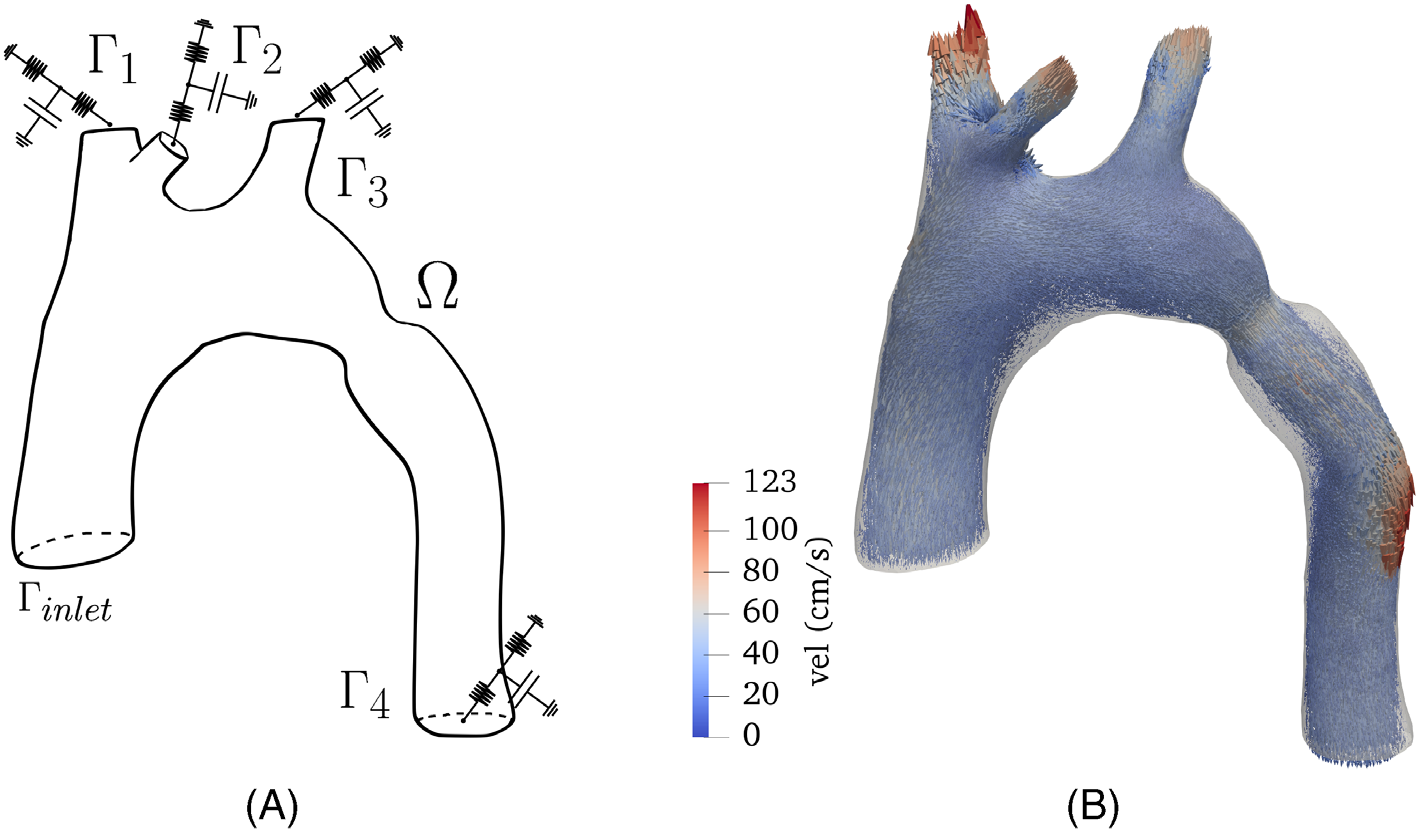
Boundary conditions for the simulation with the reference solution obtained for generate synthetic data sets. In (A) boundary conditions, (B) Velocity vector field in the reference mesh at peak systole

All testcases were created in the following way:

- A reference flow $\vec{u}$ is created using a finite element solver setup on an unstructured mesh of an aorta with a coarctation using high spatial and temporal resolutions. Physiologically relevant boundary conditions were set. The geometry with boundary conditions and resulting velocity field are shown in Figure 1. The simulation details are given in Appendix B.
- The results of the simulation where interpolated in time with a time step of 0.03 s, hence downsampled by a factor of 30.
- In order to simulate the 4D Flow MRI acquisition, a complex magnetization field was computed for every component of the reference velocity as: $M_{j}=m_{0}\;\exp\;\left(i\phi_{0}+\mathrm{i\pi}u_{j}/\text{venc}\right)$ with j=1,2,3, with $u_{1},\ldots ,u_{3}$ the time undersampled velocity fields. The value of $\phi_{0}$ was assumed to be constant in space and time and equal to $7.5\cdot 10^{-2}\;\mathrm{rad}$, and $m_{0}$ assumed constant and equal to 0.5 on the volume. The venc parameter setup will be explained in short. The background magnetization field was computed without any dependence on the velocity as $M_{0}=m_{0}\;\exp\;\left(i\phi_{0}\right)$.
- Each magnetization field $M_{0},\ldots ,M_{3}$ was interpolated into a fine voxel‐mesh with an element size of $h_{b}=1\;\mathrm{mm}$. Then, a smoothing filter on space via convolution with a Gaussian kernel with a standard deviation of $5h_{b}$ was applied, in order to take into account partial volume effects. The filtered magnetization was interpolated into a grid mesh with the desired image resolution of $2\times 2\times 2\;{\mathrm{mm}}^{3}$ around the reference mesh. Such interpolation was performed using a piecewise linear Lagrangian interpolator. Then, the four components were arranged as a multidimensional array, which will serve to create the images, denoted as $M_{0},M_{1},M_{2},M_{3}$.
- We perturbed the magnetizations adding noise and aliasing by:
  - The *venc* parameter was set lower than the peak true velocity. Therefore, when the velocity (in absolute value) exceeds the *venc*, the reconstructed velocity will be wrapped. Two *venc* parameters as the 150% and 70% of the maximum reference velocity were chosen, resulting in the values of 123 and 57 cm/s, respectively.
  - The magnetization $M_{j}$, j=0,…,3 includes Gaussian noise, having real and imaginary parts independent noise realizations with standard deviation 0.25. This results in a velocity noise with a variance of 17.73% of the maximum reference velocity, in the high *venc* case, and a variance of 11.41% in the lower *venc* case.
- Then, the 4D Flow measurements are given by

$${\vec{u}}_{\text{meas}}=\frac{\text{venc}}{\pi }\;\text{angle}\left({\tilde{M}}_{j}/{\tilde{M}}_{0}\right),$$

with / representing an element‐wise division of the arrays and ${\tilde{M}}_{j}$ corresponding to the magnetization perturbed with the noise. Here, the time step index has been omitted in the description for the sake of readability.

**FIGURE 2:**
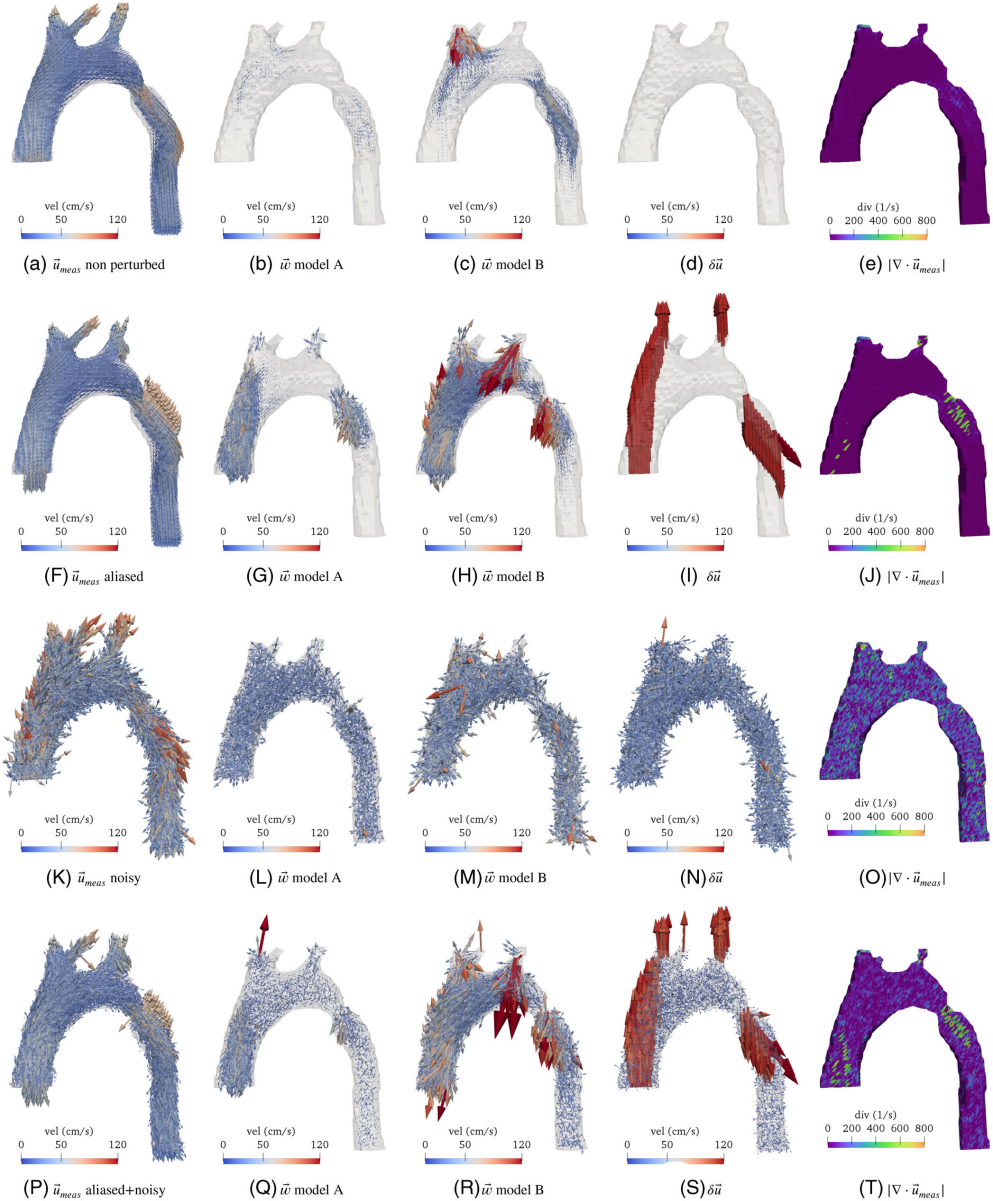
Measurement, ${\vec{w}}_{A,B}$, error and divergence field for different measurements: (A)–(E) non perturbed, (F)–(J) aliased, (K)–(O) noisy and (P)–(T) aliased and noisy

- An image mask is created from the reference simulation on the uniform‐grid mesh. Then, a new semi‐structured tetrahedral mesh following the aortic shape is created using the algorithm reported in Reference 17. The velocity is defined as a ${\mathbb{P}}_{1}$ finite element field on such mesh to visualize the results and quantify the errors to the reference solution interpolated to the same mesh. The different velocity measurements generated are shown in Figure 2, first column from left to right.

### 3.2 Results

Problem (7) was solved using synthetic velocity measurements. In all the cases, δ=1 was used for the pressure stabilization term, chosen as the smallest possible value regarding a local neighborhood insensitive to δ.

The results are shown in Figure 2 at the time of peak systole. For every data set, the two alternative formulations defined by Equations (3) and (6), referred now as *Model A* and *Model B* were tested, resulting in the fields ${\vec{w}}_{A}$ and ${\vec{w}}_{B}$, respectively. Moreover, the difference field $\delta \vec{u}={\vec{u}}_{\text{true}}-{\vec{u}}_{\text{meas}}$ is shown for comparison with $\vec{w}$. Finally, the values for $|\nabla \cdot {\vec{u}}_{\text{meas}}|$ are shown.

For the non‐perturbed measurements (first row, (a)–(d)), $\vec{w}$ grows in zones where convective effects in the flow are more significant, that is, mostly after the coarctation. It can be observed that when the measurements have no perturbations, ${\vec{w}}_{A}$ is negligible compared with ${\vec{u}}_{\text{meas}}$. The exceptions are the convection‐dominated regions where $\vec{w}$ achieves values below 30% of ${\vec{u}}_{\text{meas}}$. This could be attributed to the possibility that larger errors in the gradient of the measurements are more challenging to handle with the proposed stabilized finite element scheme. The divergence field shows similar behavior. In contrast, ${\vec{w}}_{B}$ presents very large values in a few spatial locations in spite that the measurements are perturbed only by the spatial blurring. In the pure aliased case (second row, (e)–(h)), the divergence appears to be larger after the coarctation, where aliasing is present. However, in the ascending aorta it appears to detect aliasing only in a few places. This could be related by the fact that in the case of a “straight” flow, a wrapped velocity field does maintain the divergence. In contrast, $\vec{w}$ on both models considerably grows where aliasing occurs, compared to the non‐perturbed case, but not as much as $\delta \vec{u}$. Also, a coupling among the components of the field appears, therefore $\vec{w}$ on both models, does not point exactly as $\delta \vec{u}$. Note that also not all points with aliasing are detected, most likely due to the homogenous Dirichlet boundary condition. In the case of ${\vec{w}}_{B}$, it turns out that its magnitude grows considerably in the regions where no aliasing is present, while the growth of ${\vec{w}}_{A}$ does concentrate around the aliasing.

For the pure noise case, ${\vec{w}}_{B}$ presents higher values than ${\vec{w}}_{A}$. Nevertheless, both present a similar “random” behavior as δu but with lower magnitude in the case of ${\vec{w}}_{A}$ due to its higher regularity. For the case with noise and aliasing, a combination of the two aforementioned behaviors is obtained. Note that since the velocity‐to‐noise ratio is proportional to the venc, in this case it is smaller than what is shown in the “pure noise scenario” with a larger venc. The divergence field shows again a small sensitivity to aliasing and exhibits an overall increment in the presence of noise.

Regarding the differences between both corrector fields, in summary ${\vec{w}}_{B}$ presents larger values in artifact‐free zones. ${\vec{w}}_{A}$, on the other hand, can better detect localized perturbations in the measurements. Moreover, as mentioned in Remark 5 and as it can be appreciated on Figure 2, the field $\vec{w}$ does not match δu due to its higher regularity than the perturbed measured velocities. Consequently, since the purpose of $\vec{w}$ is to simply detect faults in the data, we recommend and adopt *Model A* for the rest of the manuscript.

Additionally, a correlation study of the fields $∥\vec{w}∥$ and $|\nabla \cdot \vec{u}|$ for every case with respect to $∥\delta \vec{u}∥$ was performed. Only the results obtained by the *Model A* were used. Pearson and Spearman correlation coefficients were computed for each field at the moment of peak systole. Moreover, a rank of significant association was computed between the values of the fields using the *Maximal Information Coefficient estimator* (MI $C_{e}$) from Reference 18. All these values are shown in Tables 1, 2, 3, 4.

**TABLE 1:** Non perturbed data

	$∥\vec{w}∥$	$|\nabla \cdot {\vec{u}}_{\text{meas}}|$
Pearson coeff.	0.1493	‐0.0186
Spearman coeff.	0.2214	0.0588
MIC_e_	0.0366	0.0141

**TABLE 2:** Only aliased data

	$∥\vec{w}∥$	$|\nabla \cdot {\vec{u}}_{\text{meas}}|$
Pearson coeff.	0.5939	0.2023
Spearman coeff.	0.2510	0.1258
MIC_e_	0.2540	0.1292

**TABLE 3:** Only noisy data

	$∥\vec{w}∥$	$|\nabla \cdot {\vec{u}}_{\text{meas}}|$
Pearson coeff.	0.0026	0.0035
Spearman coeff.	0.0080	‐0.0002
MIC_e_	0.0060	0.0053

**TABLE 4:** Noisy + aliased data

	$∥\vec{w}∥$	$|\nabla \cdot {\vec{u}}_{\text{meas}}|$
Pearson coeff.	0.4911	0.2045
Spearman coeff.	0.1534	0.0681
MIC_e_	0.1303	0.0360

The $∥\vec{w}∥$ field shows better correlation with $∥\delta \vec{u}∥$ than $|\nabla \cdot {\vec{u}}_{\text{meas}}|$ in cases where aliasing is present in the measurements. Without aliasing, for both fields the correlation is much lower, having $∥\vec{w}∥$ a slightly better performance. The MI $C_{e}$ coefficient shows higher level of significance between the fields when aliasing is present as well.

## 4 NUMERICAL EXAMPLES USING REAL 4D FLOW MRI DATA

### 4.1 Experimental phantom

**FIGURE 3:**
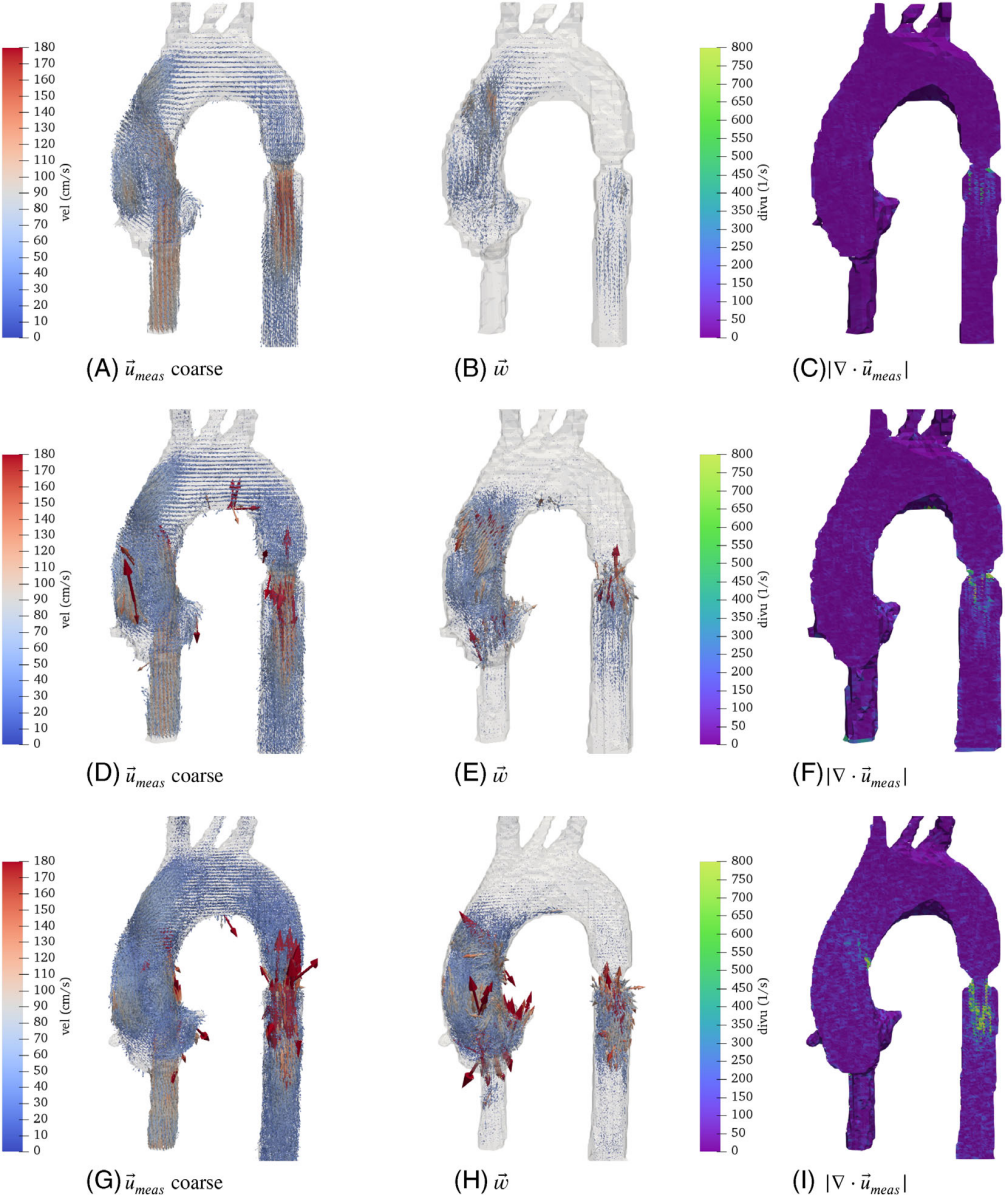
Results for aortic phantom data, for three spatial resolutions of the measurements: measurements (left), (mid) $\vec{w}$, and (right) divergence of the measurements

A realistic thoracic aortic phantom was scanned using a clinical 1.5T MR scanner (Philips Achieva, Best, The Netherlands) with a four‐element phased‐array body coil. The phantom was made of flexible silicone and a 11 mm orifice coarctation made of Technyl was placed in the descending aorta (for further details of the setup and the phantom see^19^, ^20^). A blood mimicking fluid made with 60% water and 40% glycerol (Orica Chemicals, Watkins, CO) was used in the system. The fluid has a density of $1.119\;g/{\mathrm{cm}}^{3}$, dynamic viscosity of 0.0483P and T1 value of 900 ms, which are in the range of values for human blood. The acquisition was performed with a venc of 350 cm/s and using a Cartesian sampling sequence with no *k*‐space undersampling involved. In MRI, the noise level of the image increases when decreasing the voxel size. Therefore, three isotropic voxel sizes (coarse: 2.5 mm, mid: 2.0 mm and fine: 1.5 mm) were acquired in order to investigate the results of $\vec{w}$ in front of different resolutions and SNR levels. Figure 3 shows the 4D Flow measurements together with their result for $\vec{w}$ at the moment of peak systole. Also the divergence of the measurements are included.

First, note that for all three resolutions $\vec{w}$ tends to grow in zones where the flows becomes convection dominated, and not necessarily where the velocities are high, see Figure 3A and B. For instance, in the three regions of high velocity, the inflow tube and the descending aorta post stenosis, the inflow tube presents a negligible $\vec{w}$ value, where we expect the flow to be closer to a Stokes flow. This observation agrees with the results obtained in the synthetic case. However, real measurements have an additional source of error, namely the measurement technique assumes that the velocity field is constant within the time window of observation, in this case 40 ms. This induces to larger errors in the data that are not present in the synthetic case, possibly explaining the higher $\vec{w}$ values observed in the coarse voxel case.

When decreasing the voxel size, as expected from the reduction of the signal‐to‐noise ratio, $\vec{w}$ increases with the appearance of artifacts in the measurements. This occurs in the ascending aorta, in the stenosis, and in the inferior part of the arch. For all voxel sizes the divergence shows an increment post stenosis, with much lower values in the rest of the domain. Therefore, at least in this experiment the divergence of the measurements seems not to be capable of detecting spatial regions with decreased measurement quality, it only captures the regions where the velocity is larger.

### 4.2 Subjects

Two healthy volunteers were scanned in the previously described 1.5 Tesla scanner using a 4‐channel torso coil. The local committee approved the study, and informed written consent was obtained from the participants. The acquisition parameters were: FOV in the range of 192×192×162 and $224\times 224\times 162\;{\mathrm{mm}}^{3}$, voxel resolution of $2.0\times 2.0\times 2.5\;{\mathrm{mm}}^{3}$, temporal resolution dt=34ms, venc=150cm/s, 25 cardiac phases, flip angle of 6° hand TR/TE=4.9ms/2.9ms. From the resulting data, only the aorta was segmented in order to apply our in‐house mesh generation algorithm. Afterwards, the problem was solved assuming a blood density of $\rho =1.2\;\mathrm{gr}/{\mathrm{cm}}^{3}$ and a dynamic viscosity of μ=0.035P, same values taken for the simulation with synthetic data. A study of the impact of these parameters on the solution w is presented in Appendix C.

**FIGURE 4:**
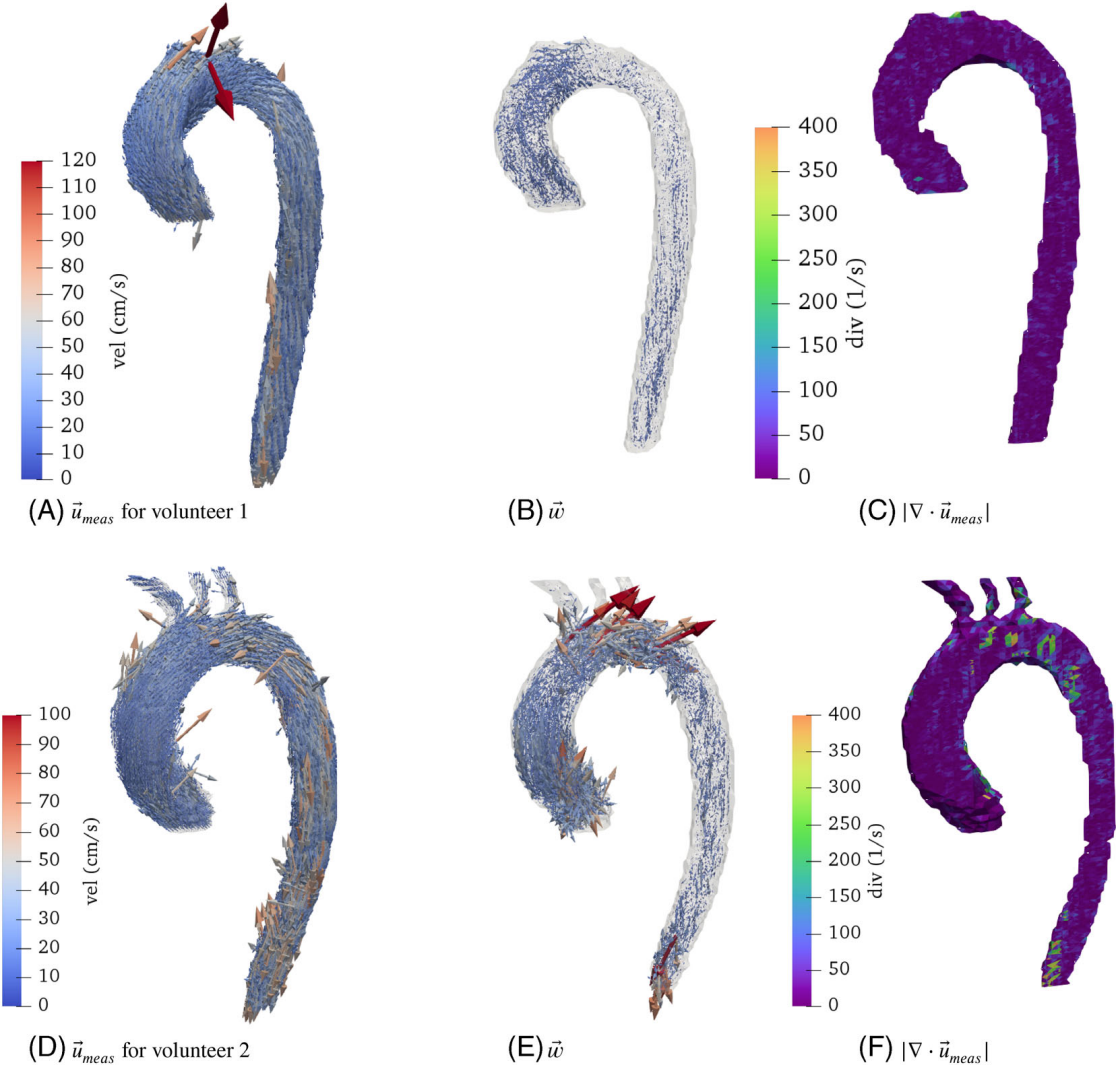
4D Flow measurements, the corresponding $\vec{w}$, velocity and divergence fields for the volunteers at the time of peak systole

Figure 4 shows the 4D Flow measurements, their divergence and the resulting $\vec{w}$ at peak systole. In all the cases $\vec{w}$ grows when flow artifacts appear in the measurements. Volunteer 1 shows a highly regular blood flow which results in a small $\vec{w}$. The divergence presents no large peaks. This suggests that the measurements are mostly perturbed by noise. Only in the ascending aorta $\vec{w}$ takes slightly higher values, probably due to larger convective effects in that region. The divergence field shows a concentrated spike on the top of the aortic arch likely to be caused by a boundary artifact, which is not detected by $\vec{w}$ due to the homogenous boundary condition.

Volunteer 2 shows a case where the velocity measurements are more perturbed, in particular on three different locations. In the ascending aorta, closer to the heart, the measurements presents discontinuities at certain locations. This is confirmed by the high values of $\vec{w}$, while the divergence seems almost not to be perturbed, compared to other regions. In the aortic arch, all three fields appear to be perturbed. A last small region with larger $\vec{w}$ and divergence values appear in the distal part of the descending aorta.

## 5 CONCLUSION

We presented a new mathematical model—including a tailor‐made discretization—to detect imperfections in full‐field velocity measurements as 4D Flow MRI can obtain it. The derived model uses data consistency with the incompressible Navier–Stokes equations, leading to a new vector field $\vec{w}$, used as an indicator to check the compatibility of the data with the physics induced by Navier–Stokes.

Synthetic data experiments show the ability of the proposed approach to detect typical artifacts in 4D Flow MRI images, such as noise and aliasing, more robustly than computing the divergence of the measurements. With real 4D Flow MRI, we showed how $\vec{w}$ increases as the data quality decreases. That indicator mark regions where the data could be misrepresenting the blood flow, which is a valuable information when further flow quantification needs to be performed. In our experiments, we observed how some of these errors were not detected using the divergence as indicator.

At representative spatial and temporal resolutions of the data, computations of the proposed finite element discretization results take about 50 s without any parallelization using standard personal computers. That makes its clinical application feasible, since most of the burden lies in the blood lumen segmentation, where several flow markers are quantified. Our work still presents some limitations. Concerning the numerical scheme, more advanced discretizations could be investigated to reduce the vector values arising from the discretization itself, particularly in convection‐dominated regions. Moreover, the proposed discretization of $\vec{w}$ cannot fully capture strong discontinuities in the measurements, for example, in aliasing‐contaminated measurements, mainly due to the regularity of the solution imposed by the discrete spaces used in this work. Therefore future work could consist in investigating the application of Discontinuous Galerkin approaches.

Another limitation of this study concerns the measurement generation in the synthetic data example. The analysis did not consider variability, due to a time blurring effect after several cardiac cycles, which occurs in the MRI measurement process due to sequential filling of the frequency space, respiratory motion artifacts, among others.

## Figures and Tables

**FIGURE C1:**
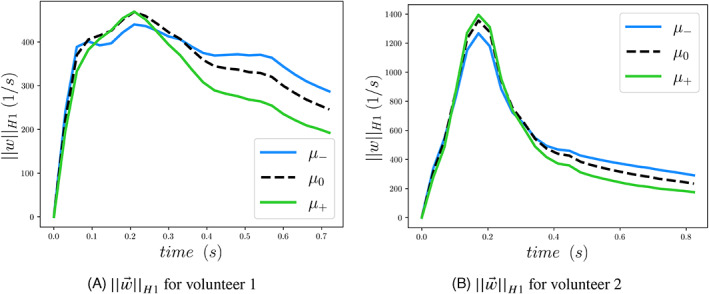
Comparison between norms of the corrector field under three different dynamic viscosity values

**FIGURE C2:**
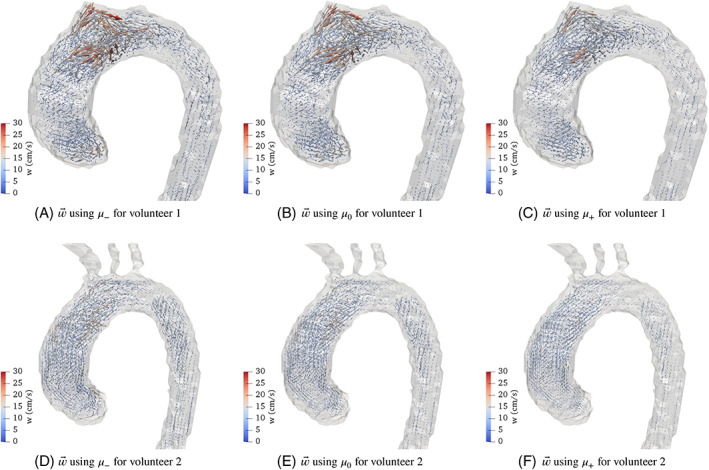
Corrector fields at t=0.6s. The arrow scale have been remained the same with respect to the scaling used in the rest of this work for a better comparison

## Data Availability

The data that support the findings of this study are available on request from the corresponding author. The data are not publicly available due to privacy or ethical restrictions.

## APPENDIX A WELL POSEDNESS ANALYSIS OF THE DISCRETE SOLUTION

### A.1 EXISTENCE AND UNIQUENESS OF THE DISCRETE SOLUTION

The purpose is now to determine if problem (7) is well‐posed by verifying if it fulfills Lax–Milgram theorem.

First, note that ${\vec{u}}_{\text{meas}}^{k}\in {\left[H^{1}\left(\Omega_{h}\right)\right]}^{3}$ for all k since the velocity data is bounded and interpolated to ${\mathbb{P}}_{1}$ finite elements.

Denote the space of the whole solution vector $W=V_{h}\times Q_{h}$, then we can prove the following results. Lemma 1
*The operator* $∥\cdot {∥}_{W}:W\to \mathbb{R}$ *defined by*

$$∥\left(\vec{v},q\right){∥}_{W}^{2}≔\beta ∥\vec{v}{∥}_{H_{1}\left(\Omega_{h}\right)}^{2}+S_{\text{press}}^{k}\left(\vec{v},q;\vec{v},q\right)$$

*is a norm on* $W=V_{h}\times Q_{h}$ *for* β>0.

*Proof*. The first term defines a norm in $V_{h}$ and the second term is a seminorm in W, so $∥\cdot {∥}_{W}$ is a seminorm in W. It remains to proof that $∥\left(\vec{v},q\right){∥}_{W}=0\Rightarrow \left(\vec{v},q\right)=\vec{0}$. Indeed, the first two terms have to be zero and therefore $\vec{v}=\vec{0}$. Therefore, ∇q is a constant and also zero‐valued since $q\in Q_{h}$. Proposition 1
*For* $\vec{v},\vec{w}\in {\mathbb{R}}^{d},C\in {\mathbb{R}}^{d\times s}$ *the following relation holds*:

$$\vec{w}\cdot C\vec{v}\leq ∥C{∥}_{\infty }∥\vec{w}{∥}_{1}∥\vec{v}{∥}_{1}\leq d∥C{∥}_{\infty }∥\vec{w}{∥}_{2}∥\vec{v}{∥}_{2}$$

*with* $∥\;{∥}_{m}$ *denoting the* $\ell^{m}$ *‐norm in* ${\mathbb{R}}^{d}$.

*Proof*. Directly from the proposition's definition. Lemma 2
*There exists* α>0 *such that*:

(A1)
$${ℬ}^{k}\left(\vec{w},p;\vec{w},p\right)>\alpha ∥\left(\vec{w},p\right){∥}_{W}^{2}$$

$\forall \;\left(\vec{w},p\right)\in W\backslash \left\{\vec{0}\right\}$ under the condition:

(A2)
$$\rho /\tau +C_{\Omega }^{-2}\mu /2-\rho 3∥\nabla {\vec{u}}_{\text{meas}}^{k}{∥}_{\infty }>0.$$

*Proof*. Using standard arguments, Poincaré's inequality and Lemma 1 the following relation holds:

$$A^{k}\left(\vec{w},p;\vec{w},p\right)+S_{\text{conv}}^{k}\left(\vec{w};\vec{w}\right)=\int_{\;\Omega }\frac{\rho }{\tau }∥\vec{w}{∥}_{2}^{2}+\mu ∥\nabla \vec{w}{∥}_{2}^{2}+\rho \left(\vec{w}\cdot \nabla \right){\vec{u}}_{\text{meas}}^{k}\cdot \vec{w}$$

$$\geq \left(\frac{\rho }{\tau }+\frac{\mu }{2C_{\Omega }^{2}}\right)∥\vec{w}{∥}_{L_{2}\left(\Omega_{h}\right)}^{2}+\rho \int_{\;\Omega }\left(\vec{w}\cdot \nabla \right){\vec{u}}_{\text{meas}}^{k}\cdot \vec{w}+\frac{\mu }{2}∥\nabla \vec{w}{∥}_{L_{2}\left(\Omega_{h}\right)}^{2}$$

$$\geq \left(\frac{\rho }{\tau }+\frac{\mu }{2C_{\Omega }^{2}}-\rho 3∥\nabla {\vec{u}}_{\text{meas}}^{k}{∥}_{\infty }\right)∥\vec{w}{∥}_{L_{2}\left(\Omega_{h}\right)}^{2}+\frac{\mu }{2}∥\nabla \vec{w}{∥}_{L_{2}\left(\Omega_{h}\right)}^{2}$$

$$\geq \beta ∥\vec{w}{∥}_{H_{1}\left(\Omega_{h}\right)}^{2}$$

with

$$\beta =\min\left(\rho /\tau +C_{\Omega }^{-2}\mu /2-\rho 3∥\nabla {\vec{u}}_{\text{meas}}^{k}{∥}_{\infty },\frac{\mu }{2}\right)>0$$

under condition (A2). By adding the remaining term $S_{\text{press}}^{k}\left(\vec{w},p;\vec{w},p\right)$, relation (A1) follows directly from the definition of the norm. Lemma 3
*There exists a constant* M>0 *such that*:

$$|{ℬ}^{k}\left(\vec{w},p;\vec{v},q\right)|\leq M∥\left(\vec{w},p\right){∥}_{W}∥\left(\vec{v},q\right){∥}_{W}$$

*for all* $\left(\vec{w},p\right),\left(\vec{v},q\right)\in W$.

*Proof*. Since

$$|B^{k}\left(\vec{w},p;\vec{v},q\right)|\leq |A^{k}\left(\vec{w},p;\vec{v},q\right)|+|S_{\text{conv}}^{k}\left(\vec{w};\vec{v}\right)|+|S_{\text{press}}^{k}\left(\vec{w},p;\vec{v},q\right)|,$$

using Cauchy–Schwarz inequality and adding the missing norm terms in $\vec{w}$ we obtain $|S_{\text{press}}^{k}\left(\vec{w},p;\vec{v},q\right)|\leq ∥\left(\vec{w},p\right){∥}_{W}∥\left(\vec{v},q\right){∥}_{W}.$

For the other terms, we can integrate the convective term by parts (hence canceling out the convective stabilization) and the pressure and divergence terms. Proceeding then using Cauchy–Schwarz:

$$|A^{k}\left(\vec{w},p;\vec{v},q\right)+S_{\text{conv}}^{k}\left(\vec{w};\vec{v}\right)|\leq \left(\frac{\rho }{\tau }+\rho ∥\nabla {\vec{u}}_{\text{meas}}^{k}{∥}_{\infty }\right)∥\vec{w}{∥}_{L_{2}\left(\Omega_{h}\right)}∥\vec{v}{∥}_{L_{2}\left(\Omega_{h}\right)}$$

$$+\rho ∥{\vec{u}}_{\text{meas}}^{k}+{\vec{w}}^{k-1}{∥}_{\infty }∥\vec{w}{∥}_{L_{2}\left(\Omega_{h}\right)}∥\nabla \vec{v}{∥}_{L_{2}\left(\Omega_{h}\right)}$$

$$+\mu ∥\nabla \vec{w}{∥}_{L_{2}}∥\nabla \vec{v}{∥}_{L_{2}}+∥\nabla p{∥}_{L_{2}\left(\Omega_{h}\right)}∥\vec{v}{∥}_{L_{2}\left(\Omega_{h}\right)}+∥\nabla q{∥}_{L_{2}\left(\Omega_{h}\right)}∥\vec{w}{∥}_{L_{2}\left(\Omega_{h}\right)}$$

$$\leq \left(\frac{\rho }{\tau \beta }+\frac{\rho }{\beta }∥\nabla {\vec{u}}_{\text{meas}}^{k}{∥}_{\infty }+\frac{\rho }{\beta }∥{\vec{u}}_{\text{meas}}^{k}+{\vec{w}}^{k-1}{∥}_{\infty }+\frac{\mu }{\beta }\right)∥\left(\vec{w},p\right){∥}_{W}∥\left(\vec{v},q\right){∥}_{W}$$

$$+\frac{1}{\sqrt{\beta }}∥\nabla p{∥}_{L_{2}\left(\Omega_{h}\right)}∥\left(\vec{v},q\right){∥}_{W}+\frac{1}{\sqrt{\beta }}∥\nabla q{∥}_{L_{2}\left(\Omega_{h}\right)}∥\left(\vec{w},p\right){∥}_{W}.$$

And to end the proof we need to bound the pressure gradient:

$$∥\nabla p{∥}_{L_{2}\left(\Omega_{h}\right)}\leq ∥\rho \left(\left({\vec{u}}_{\text{meas}}^{k}+{\vec{w}}^{k-1}\right)\cdot \nabla \right)\vec{w}+\rho \left(\vec{w}\cdot \nabla \right){\vec{u}}_{\text{meas}}^{k}+\nabla p{∥}_{L_{2}\left(\Omega_{h}\right)}$$

$$+∥\rho \left(\left({\vec{u}}_{\text{meas}}^{k}+{\vec{w}}^{k-1}\right)\cdot \nabla \right)\vec{w}+\rho \left(\vec{w}\cdot \nabla \right){\vec{u}}_{\text{meas}}^{k}{∥}_{L_{2}\left(\Omega_{h}\right)}$$

$$\leq \sqrt{\frac{\mu }{\delta h^{2}}}∥\left(\vec{w},p\right){∥}_{W}+∥\rho \left({\vec{u}}_{\text{meas}}^{k}+{\vec{w}}^{k-1}\right){∥}_{L_{2}\left(\Omega_{h}\right)}∥\nabla \vec{w}{∥}_{L_{2}\left(\Omega_{h}\right)}+∥\rho \nabla {\vec{u}}_{\text{meas}}^{k}{∥}_{L_{2}\left(\Omega_{h}\right)}∥\vec{w}{∥}_{L_{2}\left(\Omega_{h}\right)}$$

$$\leq \left(\sqrt{\frac{\mu }{\delta h^{2}}}+\frac{1}{\sqrt{\beta }}∥\rho \left({\vec{u}}_{\text{meas}}^{k}+{\vec{w}}^{k-1}\right){∥}_{L_{2}\left(\Omega_{h}\right)}+\frac{1}{\sqrt{\beta }}∥\rho \nabla {\vec{u}}_{\text{meas}}^{k}{∥}_{L_{2}\left(\Omega_{h}\right)}\right)∥\left(\vec{w},p\right){∥}_{W}.$$

Lemma 4
*There exists a constant* C>0 *such that*:

$$|{ℒ}^{k}\left(\vec{v},q\right)|\leq C∥\left(\vec{v},q\right){∥}_{W}.$$

*Proof*. We proceed using Cauchy–Schwarz inequality and adding the reminder terms to obtain the W‐norm:

$$|{ℒ}^{k}\left(\vec{v},q\right)|\leq \frac{\rho }{\tau }∥{\vec{w}}^{k-1}{∥}_{L_{2}\left(\Omega_{h}\right)}∥\vec{v}{∥}_{L_{2}\left(\Omega_{h}\right)}+|\ell^{k}\left(\vec{v},q\right)|$$

$$\leq \frac{\rho }{\tau \sqrt{\beta }}∥{\vec{w}}^{k-1}{∥}_{L_{2}\left(\Omega_{h}\right)}∥\left(\vec{v},q\right){∥}_{W}+|\ell^{k}\left(\vec{v},q\right)|.$$

The last term can be bounded as:

$$|\ell^{k}\left(\vec{v},q\right)|\leq ∥{\vec{f}}^{k}{∥}_{L_{2}\left(\Omega_{h}\right)}∥\vec{v}{∥}_{L_{2}\left(\Omega_{h}\right)}+\mu ∥\nabla {\vec{u}}_{\text{meas}}^{k}{∥}_{L_{2}\left(\Omega_{h}\right)}∥\vec{v}{∥}_{L_{2}\left(\Omega_{h}\right)}$$

$$+\sqrt{\delta \frac{h^{2}}{\mu }}∥{\vec{f}}^{k}{∥}_{L_{2}\left(\Omega_{h}\right)}\sqrt{\delta \frac{h^{2}}{\mu }}∥\left(\rho \left(\left({\vec{u}}_{\text{meas}}^{k}+{\vec{w}}^{k-1}\right)\cdot \nabla \right)\vec{v}+\rho \left(\vec{v}\cdot \nabla \right){\vec{u}}_{\text{meas}}^{k}+\nabla q\right){∥}_{L_{2}\left(\Omega_{h}\right)}$$

$$\leq \left(\frac{1}{\beta }∥{\vec{f}}^{k}{∥}_{L_{2}\left(\Omega_{h}\right)}+\frac{\mu }{\beta }∥\nabla {\vec{u}}_{\text{meas}}^{k}{∥}_{L_{2}\left(\Omega_{h}\right)}+\sqrt{\delta \frac{h^{2}}{\mu }}∥{\vec{f}}^{k}{∥}_{L_{2}\left(\Omega_{h}\right)}\right)∥\left(\vec{v},q\right){∥}_{W}.$$

Theorem 1 There exists a unique solution in W of Problem (7) under condition (A2) for all k>0.

*Proof*. Since ${\vec{w}}^{0}=\vec{0}\in {\left[H^{1}\left(\Omega \right)\right]}^{3}$, *the bilinear and linear forms fulfill the requirements of the Lax–Milgram Theorem (i.e*., Lemmas 2, 3, 4) *for* k≥1.

### A.2 TIME STABILITY OF THE DISCRETE SOLUTION

We can furthermore prove the following energy balance: Theorem 2
*For* $\left({\vec{w}}^{k},p^{k}\right)$ *solution of Problem (* *7* *), with* $\ell \left(\vec{v},q\right)=0$ *it holds*

(A3)
$$∥{\vec{w}}^{k}{∥}_{L_{2}\left(\Omega \right)}^{2}\leq ∥{\vec{w}}^{k-1}{∥}_{L_{2}\left(\Omega \right)}^{2}$$

*under the condition*

(A4)
$$\mu \geq C_{\Omega }^{2}3\rho ∥\nabla {\vec{u}}_{\text{meas}}^{k}{∥}_{\infty }$$

*Proof*. Testing (7) with $\vec{v}={\vec{w}}^{k}$ and $q=p^{k}$ and using similar arguments as in Lemma 2, it is obtained

$$\frac{\rho }{2\tau }∥{\vec{w}}^{k}{∥}_{L_{2}\left(\Omega_{h}\right)}^{2}-\frac{\rho }{2\tau }∥{\vec{w}}^{k-1}{∥}_{L_{2}\left(\Omega_{h}\right)}^{2}=-\mu ∥\nabla {\vec{w}}^{k}{∥}_{L_{2}\left(\Omega_{h}\right)}^{2}-\int_{\;\Omega }\left(\rho \left({\vec{w}}^{k}\cdot \nabla \right){\vec{u}}_{\text{meas}}^{k}\right)\cdot {\vec{w}}^{k}$$

$$-\frac{\rho }{2\tau }∥{\vec{w}}^{k}-{\vec{w}}^{k-1}{∥}_{L_{2}\left(\Omega_{h}\right)}^{2}-S_{\text{press}}^{k}\left({\vec{w}}^{k},p^{k};{\vec{w}}^{k},p^{k}\right),$$

where the two first terms in the right‐hand‐side come from the continuous problem, and the two last terms are dissipative due to the numerical scheme. Bounding the former using Poincaré's inequality and the later by zero we obtain

$$\frac{\rho }{2\tau }∥{\vec{w}}^{k}{∥}_{L_{2}\left(\Omega_{h}\right)}^{2}-\frac{\rho }{2\tau }∥{\vec{w}}^{k-1}{∥}_{L_{2}\left(\Omega_{h}\right)}^{2}\leq \left(-\mu C_{\Omega }^{-2}+3\rho ∥\nabla {\vec{u}}_{\text{meas}}^{k}{∥}_{\infty }\right)∥{\vec{w}}^{k}{∥}_{L_{2}\left(\Omega_{h}\right)}^{2},$$

which combined with condition (A4) leads to relation (A3). Remark 8 Condition (A4) is very conservative and in the test cases we observe that the condition is not satisfied. However, in our computations, we have never obtained instabilities, even in situations with temporal jumps in the data as when velocity aliasing appears.

## APPENDIX B REFERENCE SYNTHETIC AORTIC FLOW

### B.1

Synthetic data was generated in a drawn‐aortic mesh with a mild coarctation in the descending aorta and consist of 2,752,064 tetrahedrons and 510,755 vertices. An incompressible Navier–Stokes problem was solved with a monolithic formulation with ${\mathbb{P}}_{1}/{\mathbb{P}}_{1}$ finite elements and with Temam and PSPG stabilizations. The boundary conditions were set as follows. At $\Gamma_{\text{inlet}}$ the following velocity profile was imposed:

$${\vec{u}}_{\text{inlet}}=u_{\text{stokes}}\;f\left(t\right)\;\vec{n},$$

where $u_{\text{stokes}}$ corresponds to a profile resulting from solving a transient Stokes problem with a non‐homogeneous Neumann boundary condition on $\Gamma_{\text{inlet}}$ in the same geometry, while $f\left(t\right)$ is a given waveform defined as

$$f\left(t\right)=\left(\begin{array}{c} \sin\left(\frac{\mathrm{\pi t}}{T}\right)\;\text{if}\;t\leq T\;\\ \frac{\pi }{T}\left(t-T\right)e^{-\kappa \left(t-T\right)}\;\text{if}\;T_{c}>t>T. \end{array}\right.$$

Here, T gives the opening time of the heart valve, $T_{c}$ the total duration of the cardiac cycle and 1/κ represents the typical time for the closing of the valve. Then, a non‐slip condition for the velocity at the walls $\Gamma_{\text{wall}}$ was imposed. Finally, a three‐element Windkessel boundary condition for the rest of the outlets was applied, that is:

$$\mu \frac{\partial \vec{u}}{\partial n}-p\vec{n}=-P_{\ell }\left(t\right)\vec{n}\;\mathrm{on}\;\Gamma_{w,\ell },\;\ell =1,\ldots ,K$$

where K denotes the number of outlets. The term $P_{\ell }\left(t\right)$ is computed by solving the equations:

(B5)
$$\left(\begin{array}{c} P_{\ell }=R_{p,\ell }\;Q_{\ell }+\pi_{\ell }\;\\ Q_{\ell }=\int_{\Gamma_{\ell }}\vec{u}\cdot \vec{n}\;\\ C_{d,\ell }\frac{d\pi_{\ell }}{\mathrm{dt}}+\frac{\pi_{\ell }}{R_{d,\ell }}=Q_{\ell }, \end{array}\right.$$

where $R_{p,\ell }$ and $R_{d,\ell }$ represent the resistance of the vasculature proximal and distal to $\Gamma_{\ell }$, respectively, and $C_{d,\ell }$ the compliance of the distal vessels. The Windkessel constants $\left(R_{p},C,R_{d}\right)$ were tuned by hand in order to have a standard physiological flow regime to achieve approximately 70%/30% split in the peak flow rate between the descending aorta and supra‐aortic branches.^19^, ^21^

For the physical parameters of the fluid and the constants of the Windkessel models see Table B1. Additionally, blackflow stabilization was added in every outlet of the system.^22^

**TABLE B1:** Physical parameters and numerical values of the three‐element Windkessel parameters for every outlet

Parameters	Value
$\rho \;\left(\mathrm{gr}\cdot {\mathrm{cm}}^{3}\right)$	1.2
$\mu \;\left(P\right)$	0.035
$U\;\left(\mathrm{cm}\cdot s^{-1}\right)$	75
$T_{c}\;\left(s\right)$	0.80
$T\;\left(s\right)$	0.36
$\kappa \;\left(s^{-1}\right)$	70

	$\Gamma_{1}$	$\Gamma_{2}$	$\Gamma_{3}$	$\Gamma_{4}$
$R_{p}\;\left(\mathrm{dyn}\cdot s\cdot {\mathrm{cm}}^{-5}\right)$	480	520	520	200
$R_{d}\;\left(\mathrm{dyn}\cdot s\cdot {\mathrm{cm}}^{-5}\right)$	7200	11520	11520	4800
$C\;\left({\mathrm{dyn}}^{-1}\cdot {\mathrm{cm}}^{5}\right)$	$4\cdot 10^{-4}$	$3\cdot 10^{-4}$	$3\cdot 10^{-4}$	$4\cdot 10^{-4}$

The initial conditions are set as ${\vec{u}}^{0}=0$ and $\pi_{\ell }^{0}=85\;\text{mmHg}$ for ℓ=1,…,K, which correspond to approximately the periodic state of the 3D‐0D system. The simulation was performed with a total time of $T_{f}=0.8\;s$ with a timestep of dt=0.001s.

## APPENDIX C VISCOSITY SENTIVITY STUDY

### C.1

In order to know how sensitive is the corrector field $\vec{w}$ to variations of the dynamic viscosity parameter μ, we computed two fields using the values $\mu_{-}=0.0175\;P$ and $\mu_{+}=0.070\;P$ with the 4D Flow data set acquired for the healthy volunteers. These values are in a 50% range of reference value for human blood used in this work ($\mu_{0}=0.035\;P$), more concretely, we computed the $H_{1}$‐norm time curves of the corrector field over time, which are shown in Figure C1. Note that, as the second volunteer has relatively higher velocities, the effect of the viscosity will be “hidden” by the dominance of the convective forces within the fluid.

It can be seen that higher differences start to appear during *diastole*, when the Reynolds number are relatively small compared with the starting of the cycle. This can be explained by the dominance of drag forces in a low velocity regime. In Volunteer 1, the relative difference of the $H_{1}$‐norm is no more than a 15% of the values obtained using $\mu_{0}$. The latter can be confirmed from the Figure C2, in which the fields are shown at t=0.6s, corresponding to the time instant where differences are maximal. In conclusion, within a reasonable interval of viscosity values no major differences in the corrector fields are observed. Therefore, we can safely adopt the reference value $\mu_{0}=0.035\;P$.
